# Establishment and characterization of six canine hepatocellular carcinoma cell lines

**DOI:** 10.3389/fvets.2024.1392728

**Published:** 2024-05-21

**Authors:** Ja Young Lee, Kieun Bae, Jung-Hyun Kim, Hyun-Jung Han, Hun-Young Yoon, Kyong-Ah Yoon

**Affiliations:** ^1^Department of Veterinary Biochemistry, College of Veterinary Medicine, Konkuk University, Seoul, Republic of Korea; ^2^KU Animal Cancer Center, Konkuk University Veterinary Medical Teaching Hospital, Seoul, Republic of Korea; ^3^Department of Veterinary Internal Medicine, College of Veterinary Medicine, Konkuk University, Seoul, Republic of Korea; ^4^Department of Veterinary Emergency and Critical Care, College of Veterinary Medicine, Konkuk University, Seoul, Republic of Korea; ^5^Department of Veterinary Surgery, College of Veterinary Medicine, Konkuk University, Seoul, Republic of Korea

**Keywords:** dog, canine hepatocellular carcinoma, cancer cell lines, toceranib, sorafenib

## Abstract

**Background:**

Hepatocellular carcinoma (HCC) is the most common malignant liver tumor in dogs. Although surgical resection is a major treatment option for canine HCC, there are no distinct strategies for unresectable tumor subtypes or adjuvant chemotherapy for tumors with positive margins. We aimed to establish and characterize novel HCC cell lines from canine patients.

**Methods:**

The cellular morphology, general growth features and tumorigenicity of the established cell lines were evaluated. We also examined the sensitivity of the cell lines to multi-target tyrosine kinase inhibitors (TKIs).

**Results:**

We established novel canine HCC cell lines from hepatic tumors and an additional kidney tumor of six canine patients. All cell lines showed colony forming and migratory ability. KU-cHCC-001 and KU-cHCC-001-Kidney, two cell lines exhibiting high epithelial–mesenchymal transition characteristics, showed tumorigenicity in xenografted mice. Toceranib, a veterinary TKI that targets vascular endothelial growth factor (VEGFR)/platelet-derived growth factor receptor (PDGFR)/c-kit, effectively inhibited the mitogen-activated protein kinase pathway and induced apoptosis. The established canine HCC cell lines showed greater sensitivity to toceranib than to sorafenib, a first-line treatment for human HCC targeting RAF/VEGFR/PDGFR. Sorafenib showed improved anti-tumor effects when co-treated with SCH772984, an extracellular signal-regulated kinase inhibitor.

**Conclusion:**

Our study suggests new therapeutic strategies for canine HCC, and these cell lines are valuable research materials for understanding HCC tumor biology in both humans and dogs.

## 1 Introduction

Hepatocellular carcinoma (HCC) was the sixth most common cancer and third most common cause of cancer-related deaths in humans worldwide in 2020 (1, 2). Due to its aggressiveness and therapeutic limitations, the latest estimates predict that there will be >1.3 million annual deaths due to liver cancer globally by 2040 (3). The major exogenous risk factors for HCC are high rates of infection with hepatitis B and C viruses. Obesity, type 2 diabetes, alcohol consumption, and tobacco smoking are major contributors to the increasing incidence of HCC (4, 5). The precise molecular features of HCC, which have been explored in multi-omics studies, have revealed genomic alterations and potential therapeutic targets. Frequent alterations in the p53; catenin beta 1 (CTNNB1); splicing factor 3b subunit 1; SWI/SNF-related, matrix-associated, actin-dependent regulator of chromatin, subfamily a, member 4; and telomerase reverse transcriptase genes have been identified, and Wnt signaling and immune checkpoint proteins, such as programmed cell death ligand 1 and cytotoxic t-lymphocyte associated protein 4, have been suggested as prospective targets (6–8).

Although the prevalence of liver tumors in companion animals is relatively low (<1%), HCC is the most common malignant liver tumor in dogs (9, 10). A Japanese research group reported age as a risk factor for canine HCC and hyperadrenocorticism as a concurrent disorder in dogs with HCC. Welsh Corgis and Beagles are breeds predisposed to HCC (10). The prognosis of canine HCC varies according to morphological subtype. Massive, solitary HCC has a favorable prognosis after surgical treatment, with a low metastatic rate and few affected lobes (11). In contrast, nodular and diffuse HCCs are difficult to be amenable to surgical resection and involve multiple liver lobes with high mitotic rates and metastasis (9). The major cause of canine HCC is not completely understood, although hepadnavirus DNA has been detected in dogs’ blood, as pathogenic viral factors have not yet been identified in dogs with liver tumors (9, 10, 12). However, as aging is a risk factor for canine HCC, intensive studies on spontaneous liver tumors in dogs will contribute to expanding further knowledge of HCC pathogenesis as a multistep process.

Canine HCC has genetic similarities with human HCC, demonstrating frequent somatic mutations of tumor protein 53, AT-rich interaction domain 1A gene, and CTNNB1, suggesting altered signaling pathways, such as Wnt/β-catenin, and DNA damage repair (13, 14). Analysis of the genetic and molecular features of liver cancers in dogs is remarkably insufficient compared with that in humans. By studying the molecular landscape of canine HCC, researchers can gain insights into its pathogenesis, identify potential therapeutic targets, and develop novel treatment strategies. Moreover, canine HCC serves as an important preclinical model for evaluating new therapies, such as targeted agents, immunotherapies, and gene therapies. Clinical trials in dogs with HCC can provide valuable data on treatment efficacy, safety, and potential side effects, thereby offering a bridge between laboratory research and human clinical trials. Additionally, studying canine HCC may provide a better understanding of the treatment response and resistance mechanisms, which can inform clinical decision-making and improve patient outcomes in both veterinary and human medicine.

Although surgical resection is considered the primary treatment for resectable cancers, adjuvant chemotherapy is used to eradicate the remaining cancer cells and reduce the risk of recurrence. Chemotherapeutic agents, such as doxorubicin and cisplatin, have been used in the treatment of canine HCC, but their efficacy is variable, and the application of targeted therapy remains limited for canine patients. Targeted therapies have shown promise in human medicine and are currently being investigated in veterinary oncology, including canine HCC. A multi-target tyrosine kinase inhibitor (TKI), sorafenib was the first systemic therapy approved for human HCC treatment demonstrating antiangiogenic and antiproliferative effects (8). A prospective, nonrandomized clinical trial of canine patients with advanced HCC demonstrated the clinical benefits of sorafenib, with a more prolonged survival compared with conventional metronomic chemotherapy (15).

Establishment of canine HCC cell lines provides a valuable tool for studying the biology, molecular characteristics, and potential therapeutic targets of this disease. Since the first canine HCC cell line by Boomkens et al. (16), several HCC cell lines have been used to study the expression of HCC markers and stem cell-like properties (17–20). Sphere-forming cells from a canine HCC cell line display increased stem cell marker expression and chemoresistance, similar to stem cells from human cancers (20). Although canine HCC cell lines are not as widely established as human or rodent cell lines, efforts have been made to develop and characterize canine HCC cell lines for research purposes.

In this study, we aimed to establish novel canine HCC cell lines from six patients. The cell lines were characterized by *in vitro* and *in vivo* experiments to assess their proliferation, migration, and tumorigenicity. We compared the anticancer effects of the veterinary TKIs, toceranib and sorafenib, and the molecular changes in these cell lines. The study of canine HCC can contribute to our understanding of the disease in humans, facilitating translational research efforts and benefiting both veterinary and human patients. Established canine HCC cell lines provide valuable tools for cancer research, benefiting both veterinary and human patients, for investigating the underlying molecular mechanisms, studying tumor progression, and evaluating potential therapeutic targets.

## 2 Materials and methods

### 2.1 Patients’ recruitment

Patients were recruited from companion dogs visiting Konkuk University Veterinary Medical Teaching Hospital (Seoul, South Korea) to diagnose and treat suspected liver tumors. All patients underwent surgical excision of the primary tumors and were histologically diagnosed with HCC. Surgically resected specimens were collected with the informed consent of the pet owners, with the approval of the Institutional Animal Care and Use Committee (IACUC) of Konkuk University, Seoul (KU21185 and KU22180).

### 2.2 Primary cell culture

The surgically resected tumor tissues and adjacent normal tissues were subjected to primary culture of tumor cells or their paired normal cells. Each tissue sample was minced and enzymatically dissociated into single cells using collagenase II (Life Technologies, Carlsbad, CA, United States), hyaluronidase, and Ly27632 (Sigma-Aldrich, St. Louis, MO, United States). After incubation at 37°C in shaking heat block for 30 min, the cells were filtered through a 70 μm cell strainer (BD Biosciences, San Diego, CA, United States) and then cultured in Advanced Dulbecco’s modified Eagle’s medium (DMEM)/F12 with other supplements (10% fetal bovine serum, 1X Glutamax (Gibco Laboratories, NY, United States), 1X ZellSheild (Minerva Biolabs, Berlin, Germany), 10 mM HEPES (Sigma-Aldrich), 0.4 mM N-acetyl-L-cysteine(Sigma-Aldrich)). HepG2, the human hepatocellular carcinoma cell line, was purchased from Korean Cell Line Bank (Korea) and cultured in DMEM supplemented with 10% fetal bovine serum and 1% antibiotics. All cells were maintained in a humidified atmosphere of 5% carbon dioxide at 37°C.

To authenticate all cell lines, the short tandem repeats (STRs) were analyzed using StockMarks^®^ Kits for Dogs (Applied Biosystems, Foster City, CA, United States), according to the manufacturer’s protocols. Mycoplasma infection was tested using polymerase chain reaction (PCR).

### 2.3 Growth rate and colony-forming assay

To examine the proliferation rates of the cell lines, 3 × 10^3^ cells were seeded in 96-well plates and incubated for 72 h. Areas of field covered by the cells were measured using the IncuCyte™ Live-Cell Imaging System (Essen BioScience Inc., Michigan, MI, United States) at 24 h intervals. To determine population doubling time, 4 × 10^5^ cells were plated in 6-well plates and incubated for 48 h. The cells were detached and counted daily using EVE^™^ Automated Cell Counter (NanoEntek Inc., Korea) in triplicate. Cell doubling time was calculated using the following formula: doubling time (h) = (t × log2)/(logN[t2] − logN[t1]) (t = t2 − t1 = incubation time [h], N[t2] = number of cells at time t2, N[t1] = number of cells at time t1). For colony-forming assay, 8 × 10^2^ cells were seeded in 6-well plates. Fresh media were refilled twice a week. After 2 weeks, the cells were stained with 0.5% crystal violet solution, and the relative colony-covering area (%) was examined using the ImageJ software (National Institutes of Health, Bethesda, MD, United States).

### 2.4 Migration assay

We evaluated migratory ability by monitoring cells using the IncuCyte^™^ Live-Cell Imaging System. To form a monolayer, 2 × 10^4^ cells were seeded in 96-well plates and incubated for 24 h. Wounds were made in each well using a 96-pin Wound Maker, and cell migration was monitored. Images were captured every 2 h and analyzed using the IncuCyte software.

### 2.5 Western blotting

The cells were lysed in radioimmunoprecipitation assay buffer (1X, Cell Signaling Technology, Beverly, MA, United States) supplemented with phosphatase inhibitor cocktail (1X, Cell signaling Technology), protease inhibitor (1X, Quartett, Berlin, Germany), dithiothreitol (2 mM, Thermo Inc., DE, United States), and phenylmethanesulfonyl fluoride (1 mM, Sigma-Aldrich). Protein lysates were quantified using the Bio-Rad protein assay kit (Bio-Rad Laboratories, Hercules, California, United States). Equal amounts of protein were separated by sodium dodecyl sulfate-polyacrylamide gel electrophoresis and transferred to poly(vinylidene fluoride) membranes. The membranes were blocked with 5% skim milk in Tris-buffered saline with Tween buffer for 1 h and then incubated with primary antibodies overnight. Proteins were visualized using a horseradish peroxidase-conjugated secondary antibody and enhanced chemiluminescence reagent (Bio-Rad Laboratories). Detailed information on the antibodies used for western blotting is provided in Supplementary Table S1.

### 2.6 RNA sequencing analysis

Total RNA was carried out with TRIzol reagent (Invitrogen), followed by quality assessment using the TapeStation4000 System (Agilent Technologies, Amstelveen, The Netherlands), and quantification using an ND-2000 Spectrophotometer (Thermo Inc.).

#### 2.6.1 Library preparation and sequencing

Libraries were generated from total RNA utilizing the NEBNext Ultra II Directional RNA-Seq Kit (New England BioLabs, Inc., United Kingdom). mRNA was isolated using a Poly(A) RNA Selection Kit (Lexogen, Inc., Austria), followed by cDNA synthesis and shearing according to manufacturer protocols. Illumina indices 1–12 were employed for indexing, followed by PCR enrichment. Subsequently, libraries were analyzed using a TapeStation HS D1000 Screen Tape (Agilent Technologies, Amstelveen, The Netherlands) to assess mean fragment size. Quantification was conducted using a library quantification kit and StepOne Real-Time PCR System (Life Technologies, Inc., United States). High-throughput sequencing was executed as paired-end 100 sequencing using NovaSeq 6000 (Illumina, Inc., United States).

#### 2.6.2 Data analysis

The raw sequencing data underwent quality control using FastQC (21), followed by removal of adapter and low-quality reads (<Q20) using FASTX_Trimmer (22) and BBMap (23). Trimmed reads were then aligned to the reference genome using TopHat (24). Read count data were processed using the fragments per kilobase per million reads (FPKM) + geometric normalization method with EdgeR within R (25). FPKM values were estimated using Cufflinks (26). Data analysis and visualization were conducted using ExDEGA (Ebiogen Inc., Seoul, Korea). Gene Ontology Biological Processes (GO-BP) enrichment analysis was performed by using ShinyGO 0.80 database (27).

### 2.7 Hematoxylin and eosin staining and immunohistochemistry

Subjected tumor tissues or cell pellets were fixed in 4% paraformaldehyde and embedded in paraffin. Hematoxylin and eosin (H&E) and immunohistochemical (IHC) staining were performed on 4 μm-thick slide. Antigen retrieval of formalin-fixed, paraffin-embedded samples was performed using the pressure cooker method with 0.1 M sodium citrate buffer (pH 6.0). Proteins were visualized using a biotinylated secondary antibody and 3,3′-diaminobenzidine substrate (Vector Laboratories, Burlingame, CA, United States).

### 2.8 Cytotoxicity and apoptosis assay

For the cytotoxicity assay, 1.5 × 10^4^ cells were seeded in a 96-well plate to a final confluence of 80%. The following day, the cells were treated with the vehicle control, serial dilutions, or combinations of drugs, as indicated. After 24 h, cell viability was determined using CellTiter 96^®^ AQueous Non-Radioactive Cell Proliferation Assay (MTS) (Promega, Madison, WI). The half maximal concentration (IC_50_) values were obtained using GraphPad Prism 8 (GraphPad Software Inc., San Diego, CA, United States) and nonlinear regression analysis. To examine drug effects on induction of apoptosis, 8 × 10^5^ cells were seeded in a 6-well plate. After 24 h, the cells were treated with 30 μm toceranib or sorafenib for 24 h and harvested. Subsequently, the cells were washed twice with cold phosphate-buffered saline (PBS) and resuspended in 1X Binding buffer containing annexin V-APC (BD Bioscience, Bedford, MA) and 4′,6-diamidino-2-phenylindole (DAPI) (0.2 μg/mL). After incubation for 15 min at room temperature in the dark, the cells were examined by flow cytometry (CytoFLEX Flow Cytometer, Beckman Coulter, Brea, CA, United States) and analyzed using the FlowJo software (Tree Star Inc., Ashland, OR, United States). Toceranib (SU11654) was prepared in deionized water (3 mM), sorafenib (BAY439006), and SCH772984 in dimethyl sulfoxide (100 mM, 10 mM) as stock solutions (all purchased from Selleckchem, United States). For further use, each stock was diluted in the culture medium.

### 2.9 Cell cycle analysis

Cells were treated with 5 μm sorafenib for 72 h and harvested at 24 h intervals. Collected cells were fixed in 70% ethanol overnight at 4°C and then resuspended in propidium iodide solution containing RNase A for 30 min at room temperature. Subsequently, cell cycle was assessed using flow cytometry (BD FACSLyric, BD Biosciences, MD, United States) and analyzed using the FlowJo software.

### 2.10 *In vivo* tumorigenicity

Cells (1 × 10^7^) were suspended in 100 μL of PBS and subcutaneously injected at the both sides of the flanks of 6-week-old NOD.Cg-Prkdcscid Il2rgtm1Sug/Jic (NOG) mice. Three to five mice were used per cell line. The tumor volume was measured using a caliper twice a week and then calculated using the following formula: tumor volume (mm^3^) = (A × B^2^)/2 (A = the longest diameter of the tumor, B = the shortest diameter of the tumor). The mice were euthanized when their tumor volume reached 1,500 mm^3^. All the animal experiments were conducted in accordance with the guidelines of the IACUC (KU21110 and KU22118).

### 2.11 Statistical analyses

All data were statistically analyzed using GraphPad Prism 8 (GraphPad Software Inc., San Diego, CA, United States). The tumor volume growth in the xenograft model, results of apoptosis assay and cell cycle analysis were analyzed by a two-way repeated measure analysis of variance (ANOVA) with Tukey’s *post hoc* test. Other data were analyzed by one-way ANOVA with Tukey’s *post hoc* test. Statistical significance was indicated at ^*^p < 0.05, ^**^p < 0.01, ^***^p < 0.001, and ^****^p < 0.0001.

## 3 Results

### 3.1 Six cell lines established from canine patients with hepatocellular carcinoma

**Figure 1:**
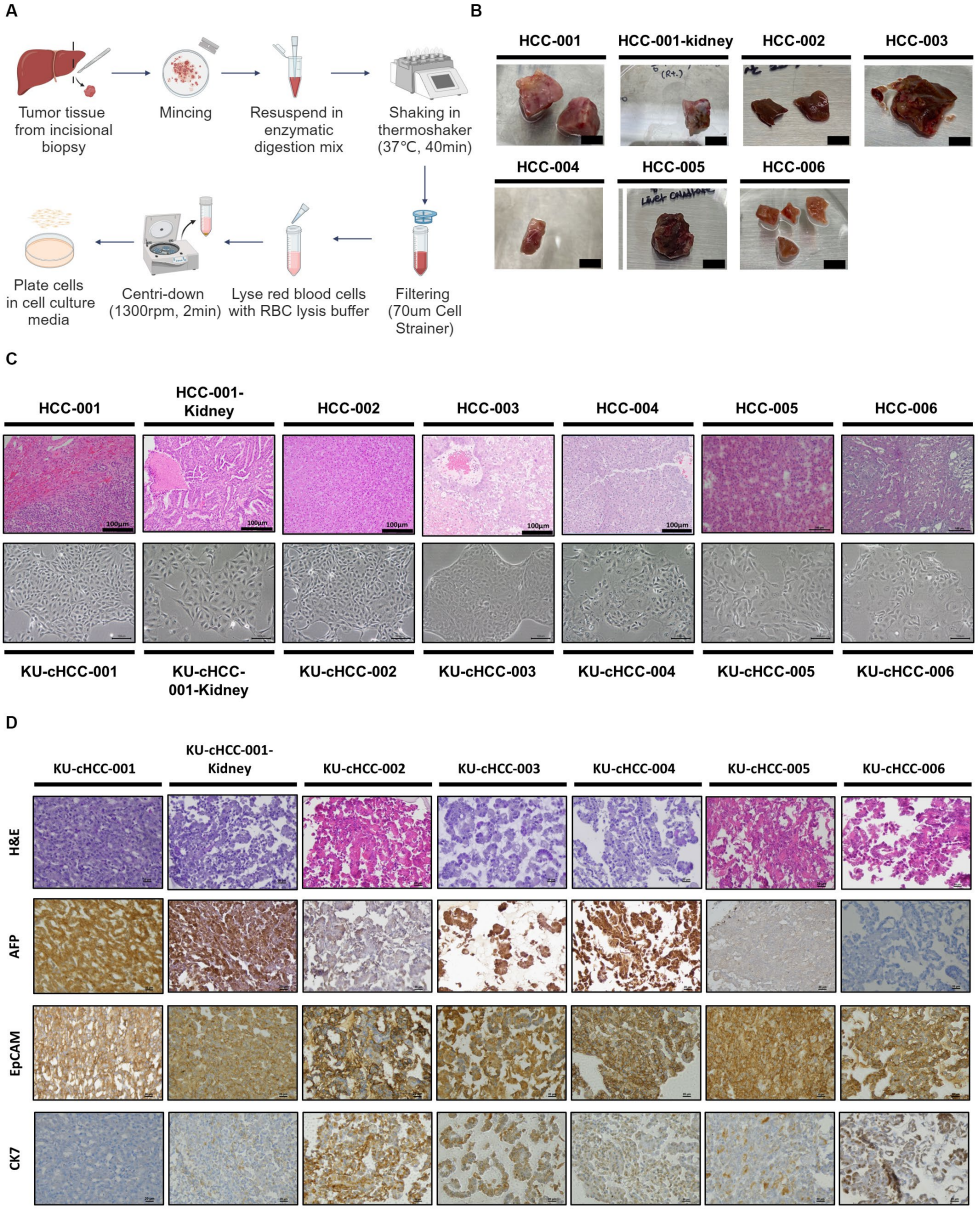
Establishment of canine hepatocellular carcinoma (HCC) cell lines. **(A)** Schematic representation of tumor tissue dissociation steps. **(B)** Primary tumor tissues resected from canine patients with HCC (scale bar = 1 cm). **(C)** Hematoxylin and eosin (H&E) slides of tumor tissues (upper panel) and morphology of their established cell lines (low panel) with scale bar of 100 μm. **(D)** Cell blocks staining with H&E and immunohistochemical staining of alpha-fetoprotein, epithelial cell adhesion molecule, and cytokeratin 7 (×400 magnification, scale bar = 20 μm).

Six canine HCC cell lines were established from canine HCC patients. All patients were pathologically diagnosed with HCC by surgical biopsy. Morphologically, two of which were nodular, whereas the others were massive. One patient had additional kidney mass, the cell line from which was designated into KU-cHCC-001-Kidney. KU-cHCC-006 was derived from patients diagnosed with combined HCC-CC (Table 1). The resected tumor tissues were minced and lysed using enzymes to dissociate them into single cells. Cultured tumor cells were initially heterogenous and were observed to be mixed with surrounding fibroblasts or immune cells. Through continuous subculturing, the epithelial tumor cells exhibited highly proliferative properties and constituted cell lines (Figures 1A,B).

**Table 1:** Demographic and clinical information of six dogs with hepatocellular carcinoma (HCC).

Identification	Breed	Sex	Age	Related medical history	Histopathological diagnosis	Localization	Resection margin	TNM stage	Morphological type
KU-cHCC-001	Maltese	SF	3	Elevated liver enzyme, Rt. Kidney mass	Carcinoma	Medial, lateral lobe	Incomplete	T4N0M1 (stage IVB)	Nodular
KU-cHCC-002	Mixed	NM	9	Elevated liver enzyme	HCC	Caudate lobe	Incomplete	T1N_M0 (stage I)	Massive
KU-cHCC-003	Dachshund	SF	10	Hemoabdomen (Tumor rupture)	HCC	Lt. medial lobe	Incomplete	T1N0M0 (stage I)	Massive
KU-cHCC-004	Maltese	NM	10	Incidental finding of hepatic mass	HCC	Lt. lateral lobe	Complete	T1N0M0 (stage I)	Massive
KU-cHCC-005	Bichon Frise	F	11	Elevated liver enzyme	HCC	Caudate, Quadrate lobe	Complete	T2N0M0 (stage II)	Nodular
KU-cHCC-006	Maltese	SF	10	Liver, adrenal, perianal mass	HCC-CC	Lt. medial lobe	Incomplete	T1N0M0 (stage I)	Massive

F: female, SF: Spayed female, NM: Neutered male, HCC: hepatocellular carcinoma, HCC-CC: combined hepatocellular-cholangiocarcinoma, TNM: tumor/node/metastasis classification scheme based on American joint committee on cancer (AJCC) cancer staging manual, 7th edition.

All the cell lines were cultured as substrate-adherent cells and grew as monolayer sheets. The morphology of cells exhibited spindle to polygonal shapes. These pleomorphic tumor cells formed a trabecular pattern as they expanded, which corresponded to the histopathological characteristics of HCCs. KU-cHCC-001 had a moderate amount of cytoplasm and smaller cell size than the other cell lines. KU-cHCC-001-Kidney, the cell line from the additional kidney mass, exhibited a similar morphology to KU-cHCC-001. Multinucleated tumor cells were frequently observed in the other cell lines, and some cells had cytoplasmic vacuoles. KU-cHCC-006 had larger, multinucleated tumor cells than the other cell lines. To confirm whether the cells retained their original morphological features, we compared cell morphology with H&E staining of the original tumor tissue. All cell lines reflected the morphology of the original tumors which they had derived from (Figure 1C).

Expressions of alpha-fetoprotein (AFP), epithelial cell adhesion molecule (EpCAM), and cytokeratin 7 (CK7) were evaluated by IHC staining of each cell block (Figure 1D). Most cell lines were positively stained for AFP, except for KU-cHCC-006. KU-cHCC-002 and KU-cHCC-005 were weakly positive than others. EpCAM showed moderate-to-strong membrane staining in all cell lines. For CK7, cell lines revealed cytoplasmic staining in a minority of cells to varying extents. KU-cHCC-001 and KU-cHCC-001-Kidney were weakly stained; KU-cHCC-002, KU-cHCC-003, KU-cHCC-004, and KU-cHCC-005 were slightly to moderately stained, whereas KU-cHCC-006 was strongly stained.

To authenticate the established cell lines, we performed an STR analysis (Supplementary Figure S1). All cell lines showed canine-specific loci in a heterozygous pattern depending on the patients from which they were derived. Additionally, the STR profile confirmed that KU-CTCC-001 and KU-CTCC-001-Kideny originated from the same patient without cross-contamination.

### 3.2 General growth characteristics of HCC cell lines

**Figure 2:**
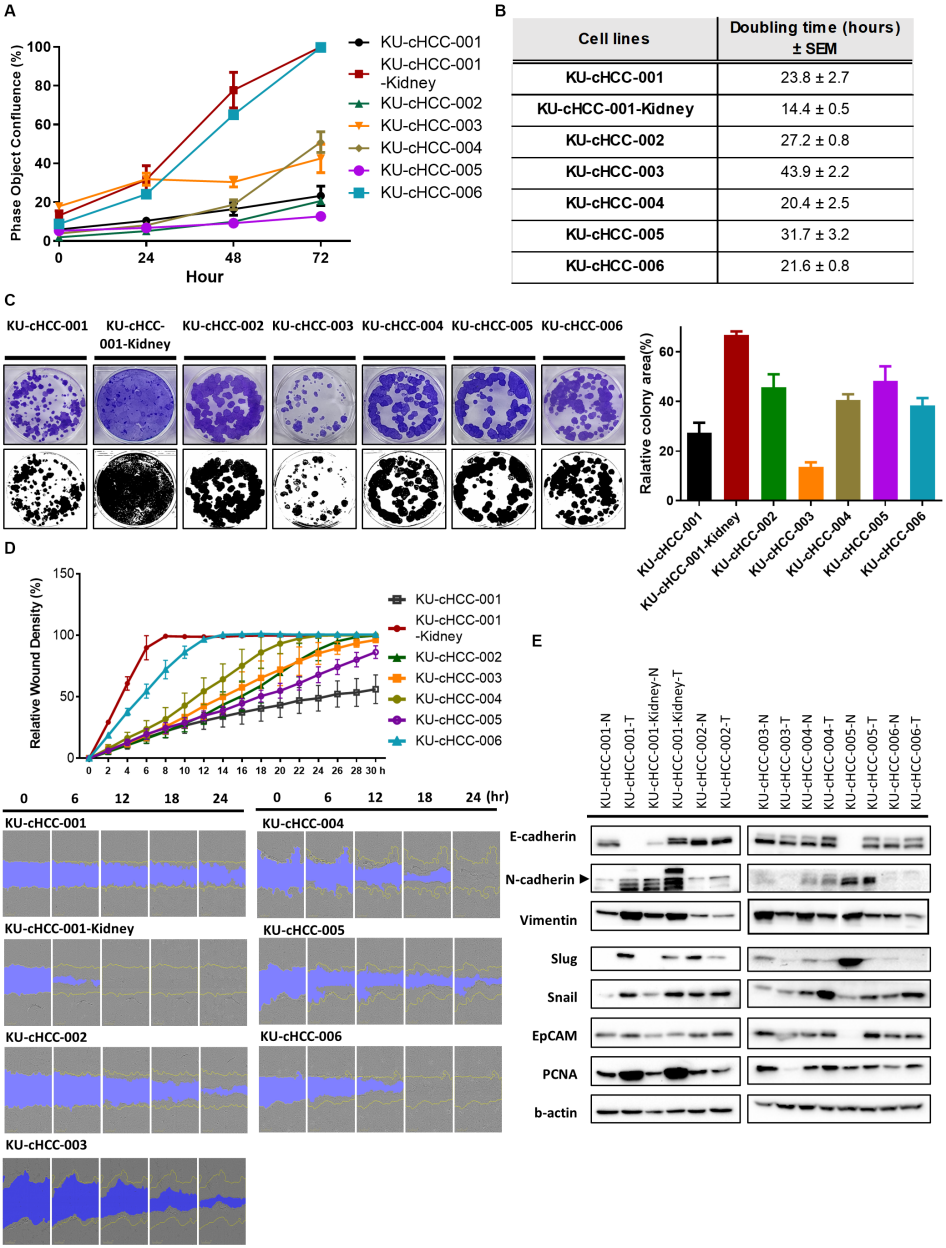
General growth characteristics of hepatocellular carcinoma (HCC) cell lines. **(A)** Comparison of proliferation rates in cell lines based on confluence. Error bars represent the standard error of the mean (SEM). **(B)** Population doubling times of each cell line with ± SEM. **(C)** Representative images of the colony-forming assay. Graph shows the colony-covering area from the triplicated experiments. Error bars represent SEM. **(D)** The wound healing ability of HCC cell lines measured by the IncuCyte^™^ Live-Cell Imaging System. Error bars represent SEM. Representative images were captured every 6 h. **(E)** Protein expression of epithelial–mesenchymal transition (EMT) markers, EpCAM and PCNA. Western blotting analysis was performed using tumor cell lines (-T) and paired normal cells (-N) derived from adjacent non-cancerous tissues. Representative images of the repeated experiments were presented. All experiments were performed in triplicate.

After a minimum of 20 passages, the proliferation rates and population doubling times of the HCC cell lines were evaluated (Figures 2A,B). Population doubling times ranged from 14.4 to 43.9 h. KU-cHCC-001-Kidney grew the fastest and had the shortest doubling time, whereas KU-cHCC-003 had the slowest doubling time. These two cell lines showed the highest and lowest colony-forming abilities, respectively (Figure 2C). No significant differences were observed between the other groups.

As the original tumors of each cell line had different clinical or histological features related to metastasis, we examined the migratory ability of the cell lines using a wound healing assay (Figure 2D). KU-cHCC-001-Kidney, KU-cHCC-004, and KU-cHCC-006 filled the wound area within 24 h, whereas KU-cHCC-001-Kidney filled the wound area the fastest. Moreover, we evaluated the protein expression of epithelial–mesenchymal transition (EMT) markers and PCNA as proliferation marker using paired normal cells, derived from adjacent non-cancerous regions (Figure 2E). KU-cHCC-001 and KU-cHCC-001-Kidney showed lower expression of E-cadherin and higher expression of mesenchymal markers, such as N-cadherin and vimentin than their normal counterparts, demonstrating that these cell lines have high EMT characteristics. PCNA was highly expressed in KU-cHCC-001, KU-cHCC-001-Kidney, KU-cHCC-004 and KU-cHCC-006, the cell lines exhibited doubling times of less than 24 h.

### 3.3 *In vivo* tumorigenicity of HCC cells

**Figure 3:**
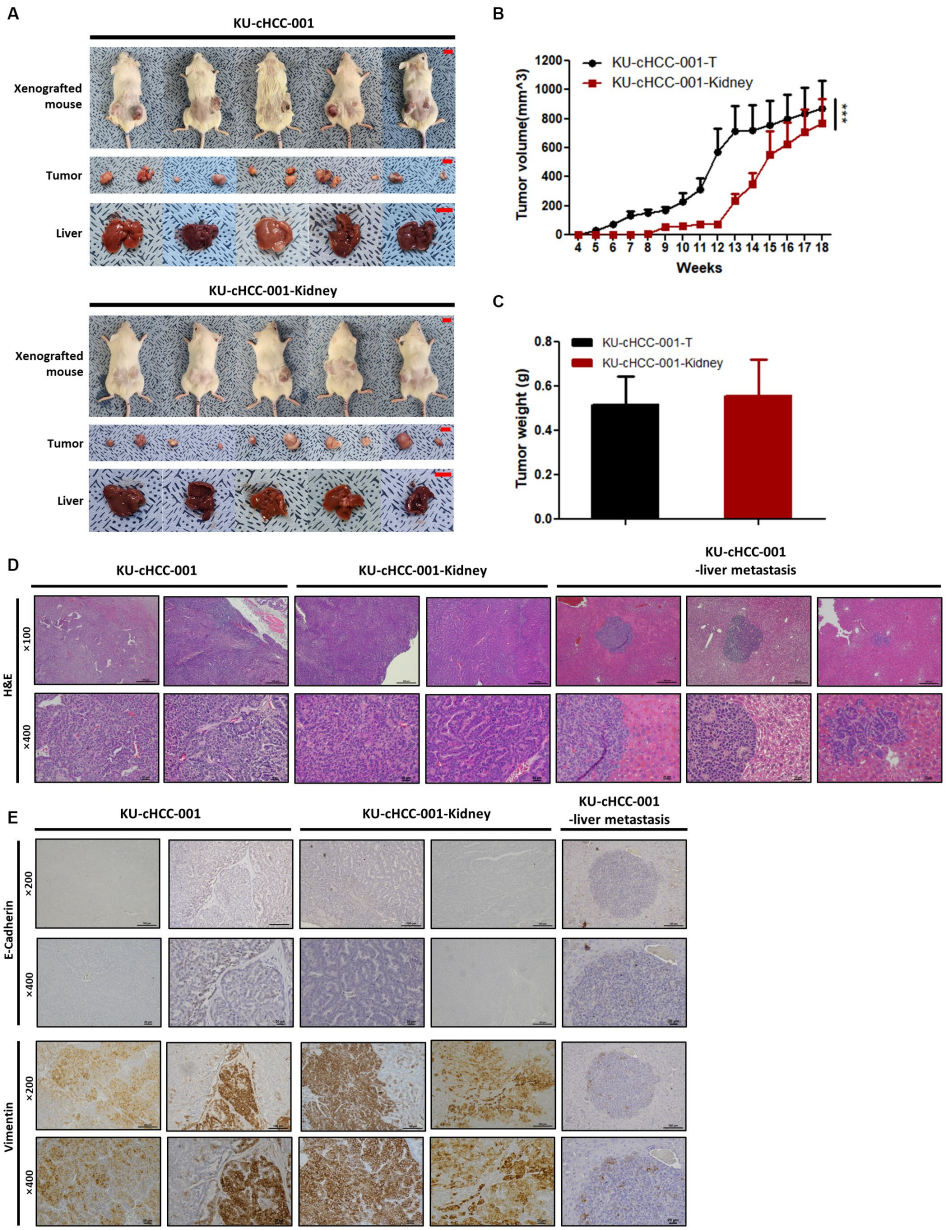
*In vivo* tumorigenicity of cell lines from KU-cHCC-001 patient. **(A)** Xenografted mice injected with KU-cHCC-001 and KU-cHCC-001-Kidney cells. Tumor specimens of subcutaneous injection sites and livers were collected after necropsy (scale bar = 1 cm). **(B)** Growth of subcutaneous tumors after injection. Error bars represent SEM. Statistical significance was determined by two-way repeated measures analysis of variance, ^***^p < 0.001. **(C)** Tumor weights measured after necropsy. Error bars represent the SEM. **(D)** Representative H&E staining of tumors and metastatic liver formed by xenograft at low (upper panel, ×100, scale bar = 200 μm) and high magnification (low panel, ×400, scale bar = 20 μm). **(E)** Immunohistochemical images of tissues presented in **(D)**. Tissue sections were stained with antibodies of EMT markers, E-cadherin and vimentin (upper panel, low magnification, ×200, scale bar = 100 μm and low panel, high magnification, ×400, scale bar = 20 μm).

The *in vivo* tumorigenicity of HCC cell lines was tested using a mouse xenograft model. The cells were subcutaneously injected into NOG mice and routinely monitored. As solid tumors formed at all inoculated sites, KU-cHCC-001 (10/10 inoculated sites) and KU-cHCC-001-Kidney (10/10) showed 100% tumorigenicity in NOG mice. Tumor formation was prominent only in mice injected with KU-cHCC-001 and KU-cHCC-001-Kidney, but not in any of the mice injected with other cell lines during monitoring period. No significant body weight loss was observed in any subject (Figure 3A; Supplementary Figure S2).

Tumors grew faster in mice injected with KU-cHCC-001 than with KU-cHCC-001-Kidney (Figure 3B). As the mice were euthanized when the tumor volume reached 1,500 mm^3^, necropsies were performed before 13 weeks in four subjects injected with KU-cHCC-001 and at 18 weeks in one subject. Liver metastasis was confirmed in mice inoculated with KU-cHCC-001 (3/5 xenografted mice) but not with KU-cHCC-001-Kidney (0/5). There was no significant difference in the tumor weight between the two cell lines (Figure 3C).

H&E staining of harvested tumor sections was performed (Figure 3D). Tumors formed by KU-cHCC-001 or KU-cHCC-001-Kidney showed similar histological features. Polygonal tumor cells with a moderate amount of cytoplasm were tightly packed in clusters or cord-like patterns, whereas necrosis was observed in the center of the tumors.

Because these two cell lines expressed upregulated vimentin and down-regulated E-cadherin as shown in Figure 2E, the tumors and metastasized livers derived from them were evaluated by IHC staining with the EMT markers (Figure 3E). Both tumors stained more positively for vimentin than for E-cadherin. The metastatic liver formed by KU-cHCC-001 injection showed vimentin-positive cells near the margins of the lesion, which were negatively stained with E-cadherin. These findings suggest that xenograft tumors and their metastatic lesions exhibit EMT properties as their originated cell lines.

### 3.4 Sensitivity of HCC cell lines to toceranib and sorafenib

We tested the sensitivity of canine HCC cell lines to toceranib (SU11654), a veterinary TKI that targets vascular endothelial growth factor (VEGFR)/platelet-derived growth factor receptor (PDGFR)/c-kit. Moreover, we compared the effect of toceranib with that of sorafenib, a multi-target TKI used as a first-line treatment for human HCC. HepG2, a nonviral human HCC cell line, was used as control.

**Figure 4:**
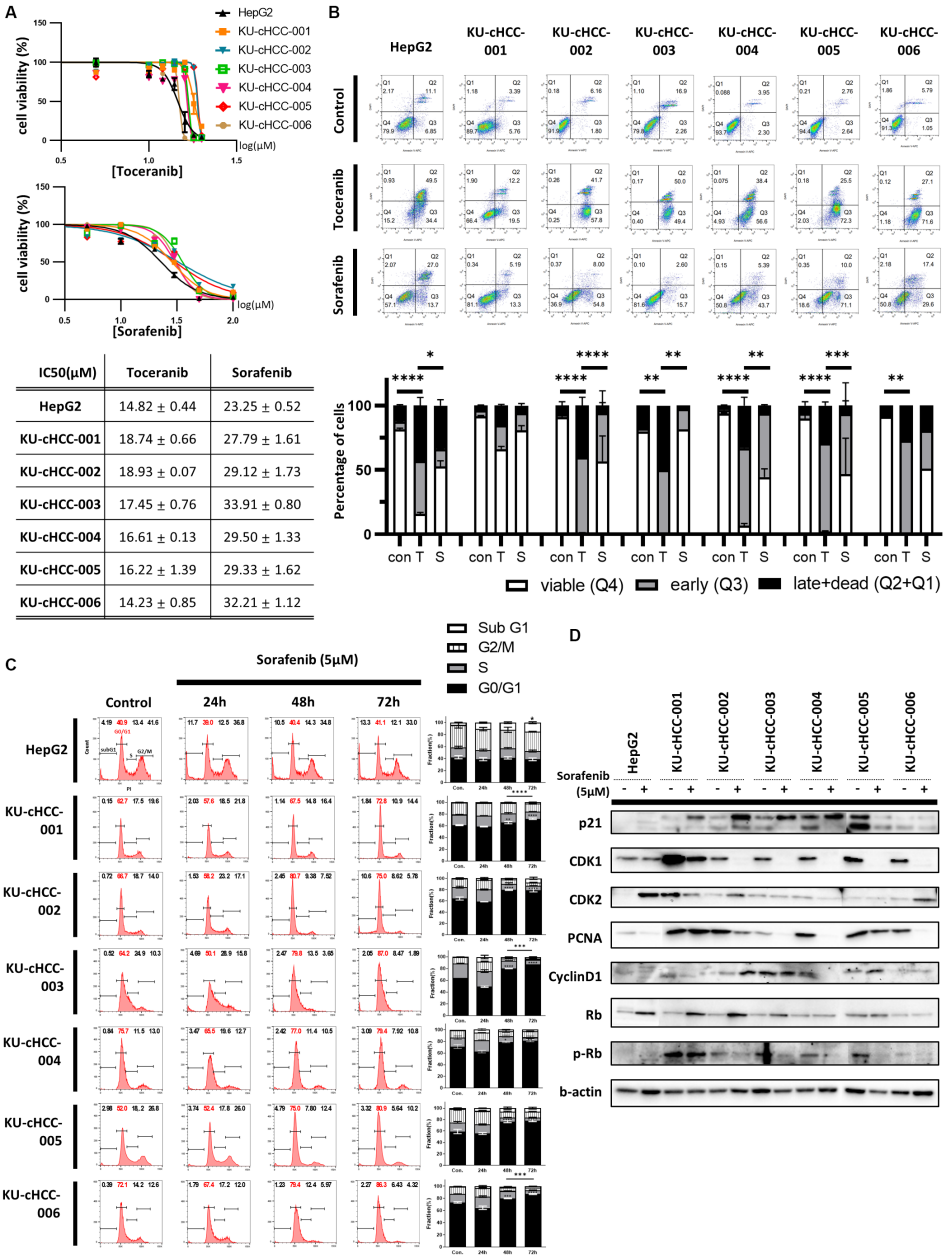
Drug sensitivity to toceranib and sorafenib. **(A)** Drug response curve and half-maximal inhibitory concentration (IC50) of toceranib and sorafenib. Error bar represents SEM. **(B)** Evaluation of apoptosis by annexin V-DAPI staining after toceranib and sorafenib treatment at 30 μM for 24 h. Percentages of cells in early apoptosis and dead cells are shown in bar graph with SEM. Statistical significance was determined by two-way ANOVA, ^*^p < 0.05, ^**^p < 0.01, ^***^p < 0.001, and ^****^p < 0.0001. **(C)** Effects of a low dose of sorafenib (5 μM) on cell cycle. Representative images of cell cycle analysis using flow cytometry are shown. The proportion of each cell phase is shown as a bar graph, with error bars representing SEM. Statistical significance, ^*^p < 0.05, ^**^p < 0.01, ^***^p < 0.001, and ^****^p < 0.0001. **(D)** Expression of proteins involved in cell cycle regulation in cells treated (+) or untreated (−) with 5 μM sorafenib.

The cell lines were treated with toceranib or sorafenib at different concentrations, and cell viability was measured. The IC_50_ values were calculated and compared between the drugs and cell lines (Figure 4A). All cell lines, along with HepG2, were more sensitive to toceranib than to sorafenib, exhibiting approximately two times lower IC_50_ values. For toceranib, KU-cHCC-006 showed the lowest IC_50_ value, and there was no significant difference between the other canine HCC cell lines. For sorafenib, higher IC_50_ were noted in all cell lines than in HepG2 cells, and there were no significant differences between the other canine HCC cell lines.

To compare the degree of apoptosis, we examined cells treated with the same concentrations of drugs using annexin V-DAPI staining and flow cytometry (Figure 4B). Toceranib caused more early and late apoptosis than sorafenib, while induced apoptosis in most cell lines compared with non-treated cells.

Since sorafenib could not induce early and late apoptosis as much as toceranib, we tried to elucidate the effects of sorafenib on cell cycle progression. Cells were analyzed after treatment with a low dose of sorafenib that did not cause severe cell death (Figure 4C). In HepG2, sorafenib treatment increased the sub-G1 fraction of the cell cycle but had no significant effect on the other phases. However, in canine HCC cell lines, sorafenib treatment increased the proportion of cells in the G1 phase and decreased the proportion of cells in the S and G2/M phases in a time-dependent manner, without significant changes in the sub-G1 fraction. We also evaluated the expression of proteins related to cell cycle regulation using western blotting (Figure 4D). p21 and Rb were upregulated in most cell lines, whereas CDK1 and PCNA were downregulated.

These findings suggest that each drug affects canine HCC cell lines. However, toceranib was more effective than sorafenib at inducing apoptosis, whereas sorafenib inhibited cell cycle progression when administered at a low dose.

### 3.5 Signaling pathways altered by toceranib and sorafenib treatment (combination effect of sorafenib with extracellular signal-regulated kinase inhibitor)

**Figure 5:**
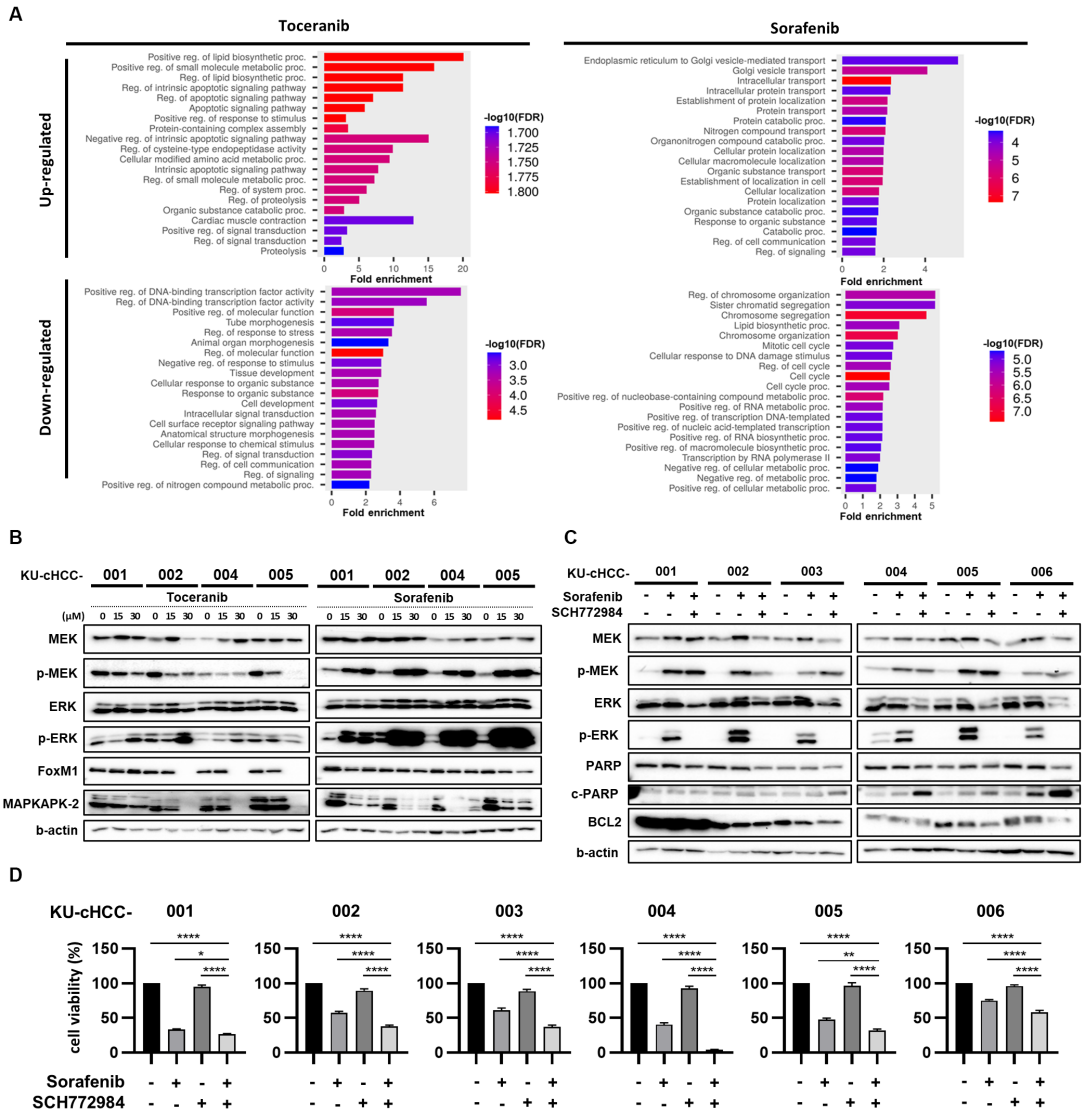
Signaling pathways altered by toceranib and sorafenib treatment [combination effect of sorafenib with an extracellular signal-regulated kinase (ERK) inhibitor]. **(A)** Gene Ontology Biological Processes (GO-BP) enrichment analysis of commonly upregulated and downregulated genes (fold change >1.5, concurrently altered in at least 3/4 cell lines examined) in KU-cHCC-001, KU-cHCC-002, KU-cHCC-004, KU-cHCC-005 after toceranib or sorafenib treatment. Top 20 pathways selected by low False Discovery Rate (FDR) are shown. **(B)** Effect of toceranib and sorafenib on MAPK pathway inhibition. Protein expression levels of MEK, ERK, FOXM1 and MAPKAPK-2 were assessed using western blot analysis. **(C)** Comparison of protein levels by sorafenib alone and in combination with an ERK inhibitor. Cells were treated with 30 μm sorafenib alone or in combination with 5 μm SCH772984 24 h. **(D)** Effects of combined treatment of sorafenib and SCH772984 on cell viability. Cell viability was measured after treatment with either one drug or both. Error bars represent SEM. Statistical significance, ^*^p < 0.05, ^**^p < 0.01, ^***^p < 0.001, and ^****^p < 0.0001.

To investigate the transcriptional changes induced by toceranib or sorafenib treatment, KU-cHCC-001, KU-cHCC-002, KU-cHCC-004, and KU-cHCC-005 were treated with each drug, and their transcriptomes were analyzed by RNA sequencing and compared to those of vehicle controls. The top 20 biological processes enriched for genes commonly upregulated or downregulated in four cell lines after drug treatment were demonstrated in Figure 5A and Supplementary Table S2. In addition, we evaluated mitogen-activated protein kinase (MAPK) pathway inhibition and the expression levels of forkhead box protein M1 (FOXM1) (28) and MAPK-activated protein kinase 2 (MAPKAPK-2) (29) using western blotting (Figure 5B). Toceranib treatment inhibited the expression of phosphorylated MAPK kinase (MEK) 1/2 (p-MEK1/2) and phosphorylated extracellular signal-regulated kinase (ERK) 1/2 (p-ERK1/2). In contrast, sorafenib treatment drastically increased the p-MEK1/2 and p-ERK1/2 levels. Both drugs downregulated FOXM1 and MAPKAPK-2 expressions. These results indicate that sorafenib, unlike toceranib, has little effect on MAPK pathway inhibition but induces ERK1/2 activation.

To investigate whether an ERK inhibitor could suppress sorafenib-induced ERK activation, we tested SCH772984, a highly selective, adenosine triphosphate-competitive ERK inhibitor of ERK1/2, in combination with sorafenib. The cell lines were co-treated with sorafenib and SCH772984, and whole-cell lysates were examined by western blotting (Figure 5C). Compared with the untreated samples, p-MEK and p-ERK were upregulated by sorafenib monotherapy in all cell lines. These activated ERK levels were abolished by co-treatment with SCH772984, whereas p-MEK1/2 levels remained unchanged.

As the combination treatment with SCH772984 markedly suppressed the induction of ERK activation by sorafenib in HCC cell lines, we further examined whether the ERK inhibitor could act synergistically with sorafenib to reduce the viability of tumor cells (Figure 5D). In all cell lines, except KU-cHCC-001, the combination treatment was more effective than sorafenib monotherapy, whereas SCH772984 alone had little effect on cell viability. The protein expression levels of apoptosis-associated markers, such as poly(ADP-ribose) polymerase (PARP) and B-cell lymphoma 2 (BCL2), were also evaluated (Figure 5C). A decrease in PARP expression was observed in the combination-treated groups of all cell lines as cleaved PARP levels increased in KU-cHCC-003, KU-cHCC-004, KU-cHCC-005 and KU-cHCC-006. BCL2 levels decreased in KU-cHCC-003, KU-cHCC-005, and KU-cHCC-006, when they were co-treated.

## 4 Discussion

HCC is the most common malignant liver tumor in dogs. The prognosis of canine HCC varies according to morphological subtype. Massive solitary HCC shows a favorable prognosis after surgical resection, whereas unresectable nodular and diffuse HCCs have a poor prognosis with limited treatment options.

In our cases, a majority of the original tumors were of the massive type (4/6 patients), and others were of the nodular type (2/6). The tumor in patient HCC-006 was diagnosed as HCC-CC, which is a rare malignant tumor with both hepatocellular and bile ductal components. The tumor of patient HCC-001 had additional kidney mass, composed of polygonal neoplastic epithelial cells with indistinct cell borders and moderate amount of cytoplasm forming tubules and small acini, and such histological features were similar to those of the liver tumor. Considering that comparable tumor cells also accumulated in the right renal vein and caudal vena cava of the same patient, the tissue of origin could not be determined owing to its disseminated nature. Regarding the aggressive nature of the original tumor, KU-cHCC-001 and KU-cHCC-001-Kidney showed 100% tumorigenicity in xenografted mice. Moreover, both cell lines exhibited EMT-associated protein expression, lower expression of E-cadherin and higher expression N-cadherin and vimentin than their normal pair cells.

Although all HCC cell lines showed colony forming and migratory capacity, only the cell lines derived from patient KU-cHCC-001, diagnosed with stage IV carcinoma, showed *in vivo* tumorigenicity. The non-tumorigenic result of the other cells may represent less aggressiveness of early-stage cancer since five patients were diagnosed in stage I or II without regional lymph node metastasis or distant metastasis as shown in Table 1. Furthermore, we tried to explain the distinct aggressive characteristics of KU-cHCC-001 cell line that exhibited liver metastasis as well as tumor formation in xenografted mice. We compared the expression pattern of EMT and cell cycle related genes by using RNA-seq data of untreated (vehicle control) cells that were shown in Figure 5A. Hierarchical clustering heatmap analysis of 84 EMT-related genes revealed that key transcription factors of EMT, such as Slug, TWIST1, ZEB1, and ZEB2, were highly expressed in KU-cHCC-001 compared to −002, −004, −005 (Supplementary Figure S3). These findings would support to explain the aggressive features of KU-cHCC-001 compared to other cell lines.

Surgical resection of tumors is a major treatment option for canine HCC; however, there are no distinct strategies for the unresectable subtype or adjuvant chemotherapy for tumors with positive margins. Systemic conventional chemotherapy has rarely been reported and has shown unsatisfactory results. One study found that seven dogs diagnosed with unresectable and treated with intravenous gemcitabine exhibited median progression-free intervals and survival times of 150 and 197 days, respectively (30). In another study, two dogs with unresectable and metastatic HCC showed no response to treatment with gemcitabine or carboplatin (31). The development of drug resistance in tumor cells is a significant challenge in the clinical management of patients with HCC, leading to treatment failure and recurrence (32). Therefore, new therapeutic approaches are required for patients with canine HCC.

In this study, we compared the anti-tumor effects of two multi-target kinase inhibitors in canine and human HCC cell lines. Our canine HCC cell lines, along with HepG2, were more sensitive to toceranib than to sorafenib, exhibiting increased apoptosis and inhibition of the MAPK pathway at the protein level. Toceranib, a veterinary TKI targeting VEGFR/PDGFR/c-kit, was originally used to treat canine mast cell tumors. However, recently, this drug has been used to treat other types of solid tumors, including HCCs (33–35). As such, previous reports have often focused on retrospective studies of clinical outcomes, and our findings contribute more to the molecular basis. In addition, toceranib exhibits potent anti-tumor effects in human HCC cell lines and cell line-derived xenograft models (36). From this perspective, toceranib is a promising chemotherapeutic option not only for canine HCCs but also for human HCCs.

On the contrary, sorafenib is a multitarget TKI targeting RAF/VEGFR/PDGFR and is used as a first-line treatment for human HCC. Nevertheless, treatment with sorafenib had little effect on MAPK pathway inhibition and rather induced the activation of ERK1/2 in our canine HCC cell lines. We then tested co-treatment with sorafenib and SCH772984, a highly selective ERK1/2 inhibitor, and confirmed that the combination treatment with SCH772984 markedly abrogated ERK activation induced by sorafenib and was more effective than monotherapy with each drug. Such combinational treatments of sorafenib with MEK/ERK inhibitors have been widely investigated in human HCCs (37–41), whereas our study is the first attempt in veterinary medicine. Thus, combination treatment with sorafenib and SCH772984 may be a new treatment approach for canine HCCs. However, as all our results concerning canine HCC sensitivity to sorafenib or toceranib were based on *in vitro* tests using patient-derived cells, these newly suggested therapeutic strategies should be supported by further clinical trials.

Furthermore, these canine HCC cell lines are valuable for comprehensive research on both human and canine HCC biology.

## 5 Conclusion

We established novel canine HCC cell lines from six primary tumor tissues and one additional kidney mass from patients with spontaneously occurring canine HCC. The two cell lines, KU-cHCC-001 and KU-cHCC-001-Kidney, exhibited strong EMT characteristics and tumorigenicity in *in vivo* xenografts. In addition, we compared the effects of two TKIs, toceranib and sorafenib, in our cell lines. Toceranib was more effective than sorafenib, causing more apoptosis, whereas sorafenib showed an improved anti-tumor effect when co-treated with SCH772984, an ERK inhibitor. These cell lines can be valuable research materials for understanding HCC tumor biology in both humans and dogs. In addition, our study sheds light on new therapeutic strategies for canine HCC that warrant further investigation.

## Data availability statement

The original contributions presented in the study are publicly available. This data can be found here: https://www.ncbi.nlm.nih.gov/geo/; GSE267624.

## Ethics statement

The animal studies were approved by Institutional Animal Care and Use Committee (IACUC) of Konkuk University, Seoul. The studies were conducted in accordance with the local legislation and institutional requirements. Written informed consent was obtained from the owners for the participation of their animals in this study.

## Author contributions

JL: Writing – original draft, Investigation, Formal analysis, Data curation. KB: Writing – original draft, Validation, Investigation. J-HK: Writing – review & editing, Validation, Supervision. H-JH: Writing – review & editing, Supervision, Resources. H-YY: Writing – review & editing, Supervision, Resources. K-AY: Writing – review & editing, Writing – original draft, Supervision, Investigation, Funding acquisition, Conceptualization.
