# Supplementary material for: Establishment and characterization of six canine hepatocellular carcinoma cell lines
Source: Front Vet Sci. 2024 May 21;11:1392728. doi: 10.3389/fvets.2024.1392728 (PMC11150866; doi:10.3389/fvets.2024.1392728)
Supplement: Supplementary file 1 [file Table_1.pdf]

Antibodies	Company	Cat. Number	Experiment
**AFP**	Origene	TA501782	IHC
**EpCAM**	Abcam	Ab71916	WB, IHC
**CK7**	Invitrogen	MA1-06315	IHC
**E-cadherin**	Cell Signaling Technology	3195	WB, IHC
**N-cadherin**	Cell Signaling Technology	13116	WB
**Vimentin**	Cell Signaling Technology	5741	WB, IHC
**Slug**	Cell Signaling Technology	9585	WB
**Snail**	Cell Signaling Technology	3895	WB
**PCNA**	Cell Signaling Technology	13110	WB
**P21**	Proteintech	10355-1-AP	WB
**CDK1**	BD biosciences	610038	WB
**CDK2**	BD biosciences	610146	WB
**Cyclin D1**	BD biosciences	556470	WB
**Rb**	Santa Cruz Biotechnology	sc-102	WB
**p-RB**	Santa Cruz Biotechnology	sc-514031	WB
**ERK**	Cell Signaling Technology	4696	WB
**p-ERK**	Cell Signaling Technology	4370	WB
**MEK**	Cell Signaling Technology	4694	WB
**p-MEK**	Cell Signaling Technology	9154	WB
**MAPKAPK-2**	Santa Cruz Biotechnology	sc-393609	WB
**FoxM1**	Santa Cruz Biotechnology	sc-376471	WB
**b-actin**	Sigma-Aldrich	A1978	WB
**Rabbit IgG**	Cell Signaling Technology	7074	WB
**Mouse IgG**	Cell Signaling Technology	7076	WB

Supplementary Table 1. Antibodies used in western blot and immunohistochemistry staining.
