# Supplementary material for: Establishment and characterization of six canine hepatocellular carcinoma cell lines
Source: Front Vet Sci. 2024 May 21;11:1392728. doi: 10.3389/fvets.2024.1392728 (PMC11150866; doi:10.3389/fvets.2024.1392728)
Supplement: Supplementary file 3 [file Image_1.pdf]

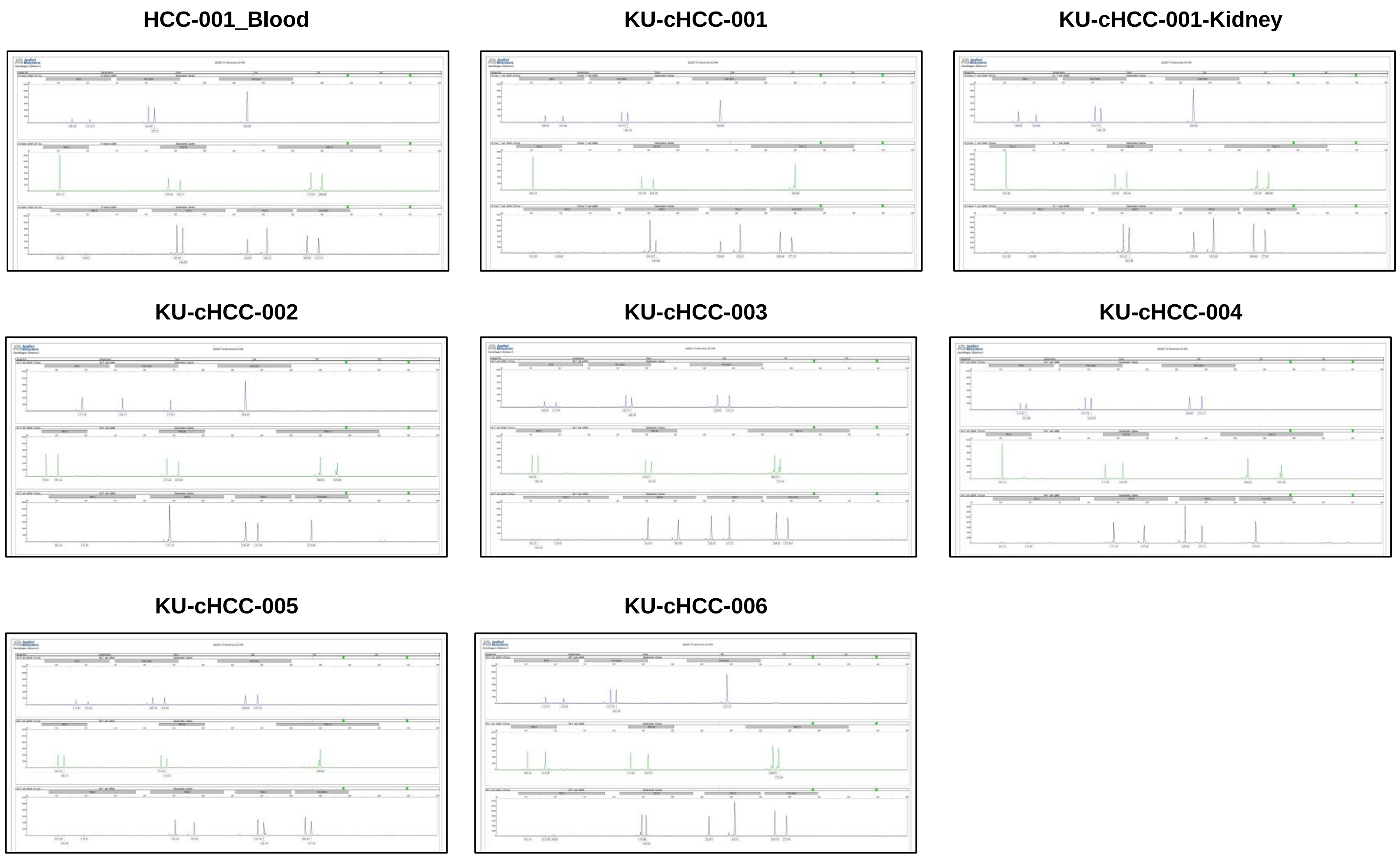

Supplementary Figure 1. STR analysis to authenticate cell lines. All cell lines showed canine-specific loci in a heterozygous pattern depending on the patients from which they were derived. The profile conformed that KU-CTCC-001 and KU-CTCC-001-Kideny were originated from the same patient without cross-contamination. HCC-001-Blood represents blood sample derived from patient HCC-001.

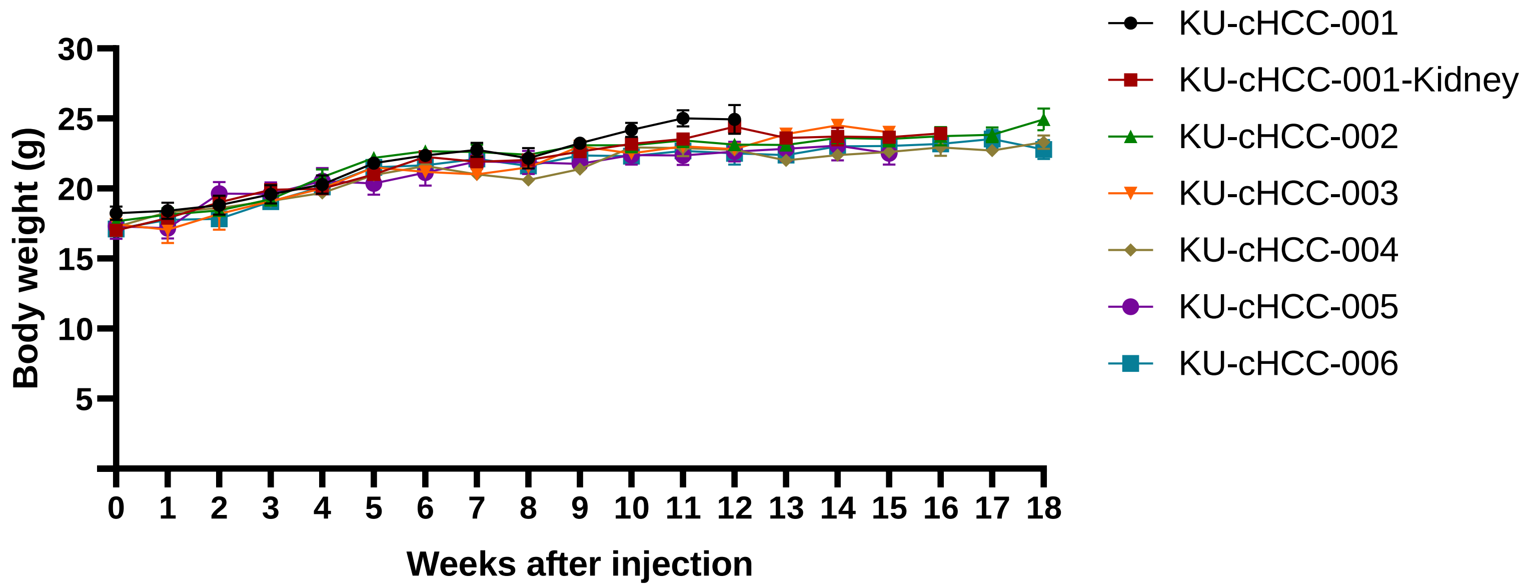

Supplementary Figure 2. Body weight of xenograft mice after inoculation of canine HCC cell lines. Tumor growth at the injection site was not observed in the mice inoculated with KU-cHCC-002, KU-cHCC-003, KU-cHCC-004, KU-cHCC-005 and KU-cHCC-006. Mice with no tumor growth were euthanized at 16 or 18 weeks after injection. No significant body weight loss was observed. Error bars represent the standard error of the mean (SEM).

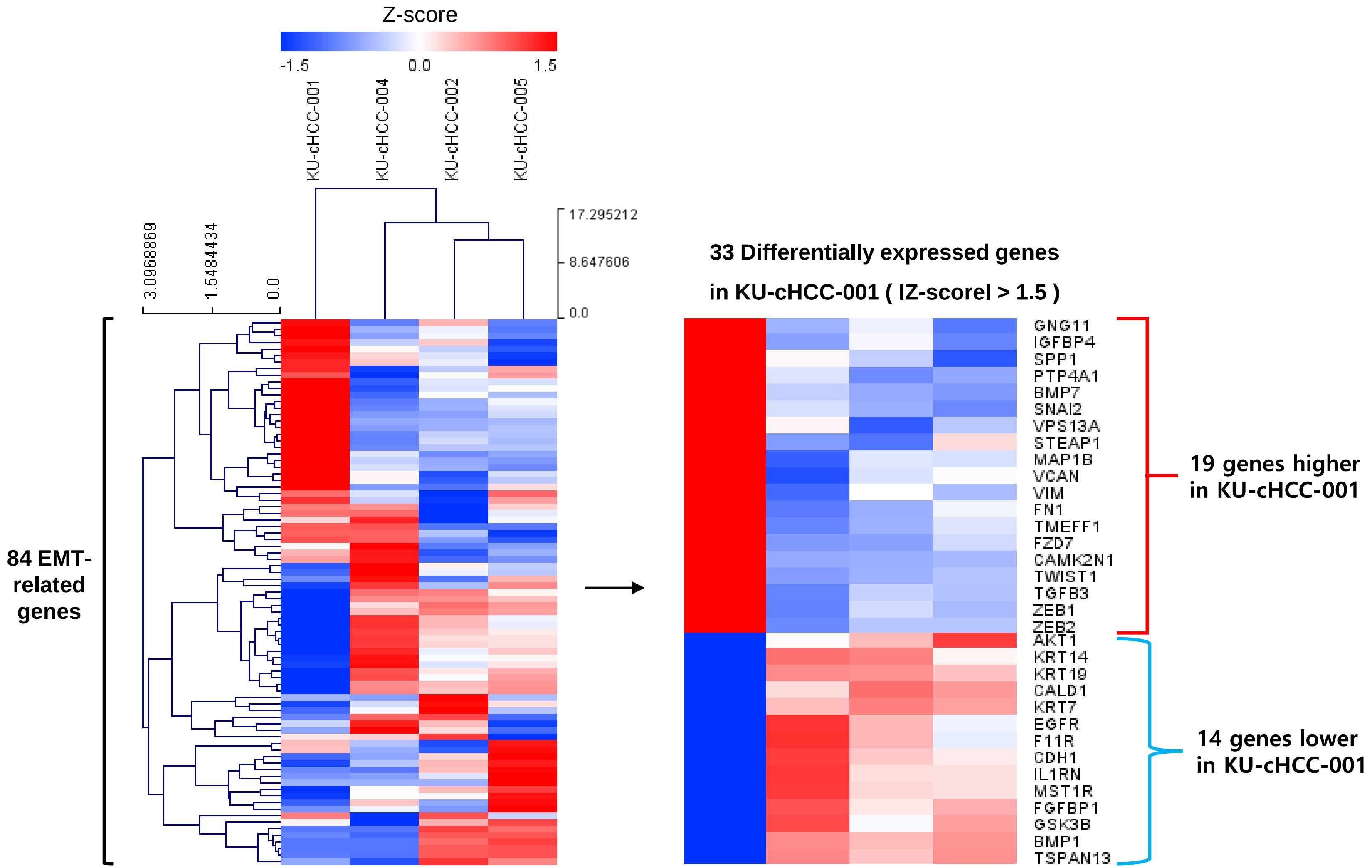

Higher expression in KU-cHCC-001	Lower expression in KU-cHCC-001
CAMK2N1	CDH1
FN1	FGFBP1
FZD7	IL1RN
GNG11	KRT19
IGFBP4	MST1R
MAP1B	TSPAN13
PTP4A1	AKT1
SNAI2 (Slug)	BMP1
STEAP1	CALD1
TGFB3	EGFR
TMEFF1	F11R
TWIST1	GSK3B
VCAN	KRT14
VIM (Vimentin)	KRT7
VPS13A	
ZEB1	
ZEB2	
BMP7	
SPP1	

Supplementary Figure 3. Hierarchical clustering heatmap analysis of 84 EMT-related genes using RNA-seq data of control cells (KU-cHCC-001, KU-cHCC-002, KU-cHCC-004 and KU-cHCC-005). For the identification of 84 EMT-related genes, a list of genes previously implicated in EMT* was employed. Z-score of each gene was calculated using normalized data (Log2(FPKM+Geometric Normalized RC+1)). Hierarchical clustering heatmap analysis was conducted by Euclidean distance metrics, using MeV software* (2) (ver. 4.9.0). Differentially expressed genes (|Z-score| > 1.5) of KU-cHCC-001 were listed (right).

*http://www.sabiosciences.com/rt_pcr_product/HTML/PAHS-090Z.html

*Howe EA, Sinha R, Schlauch D, Quackenbush J "RNA-Seq analysis in MeV." Bioinformatics 2011; 27(22):3209-10 https://doi.org/10.1093/bioinformatics/btr490
